# Asymmetric Rydberg blockade of giant excitons in Cuprous Oxide

**DOI:** 10.1038/s41467-021-23852-z

**Published:** 2021-06-11

**Authors:** Julian Heckötter, Valentin Walther, Stefan Scheel, Manfred Bayer, Thomas Pohl, Marc Aßmann

**Affiliations:** 1 Experimentelle Physik 2, Technische Universität Dortmund, Dortmund, Germany; 2 Center for Complex Quantum Systems, Department of Physics and Astronomy, Aarhus University, Aarhus C, Denmark; 3 ITAMP, Harvard-Smithsonian Center for Astrophysics, Cambridge, MA USA; 4 Institut für Physik, Universität Rostock, Rostock, Germany; 5 Ioffe Institute, Russian Academy of Sciences, St. Petersburg, Russia

**Keywords:** Electronic properties and materials, Semiconductors

## Abstract

The ability to generate and control strong long-range interactions via highly excited electronic states has been the foundation for recent breakthroughs in a host of areas, from atomic and molecular physics to quantum optics and technology. Rydberg excitons provide a promising solid-state realization of such highly excited states, for which record-breaking orbital sizes of up to a micrometer have indeed been observed in cuprous oxide semiconductors. Here, we demonstrate the generation and control of strong exciton interactions in this material by optically producing two distinct quantum states of Rydberg excitons. This is made possible by two-color pump-probe experiments that allow for a detailed probing of the interactions. Our experiments reveal the emergence of strong spatial correlations and an inter-state Rydberg blockade that extends over remarkably large distances of several micrometers. The generated many-body states of semiconductor excitons exhibit universal properties that only depend on the shape of the interaction potential and yield clear evidence for its vastly extended-range and power-law character.

## Abstract (web summary)

Previous research showed the existence of Rydberg excitons with large principle quantum numbers in Cu_2_O. Here, by using two-color pump-probe optical spectroscopy, the authors demonstrate the generation and control of long-range correlations between these giant Rydberg excitons, leading to exciton blockade.

## Introduction

Immersing an exciton into a many-body state of another species gives rise to a number of fascinating phenomena, ranging from the formation of polarons^1^ to the emergence of spinor interactions in semiconducting materials^2,3^. However, reaching and probing the regime of strong interactions has remained challenging, whereby experimental signatures of interactions are often confined to measurements of spectral line shifts due to short ranged collisional interactions. On the other hand, the strong interactions between Rydberg excitons can act over large distances^4^, which suggests new opportunities for probing the fundamental interactions between excitons.

In the past decades, the emergence of strong long-range interactions between highly excited Rydberg states was realized in a variety of atomic and molecular systems, ranging from trapped atoms^5^ and ions^6^ to Rydberg molecules^7,8^. In the field of quantum optics, the strong mutual interactions between Rydberg states can mediate enhanced optical nonlinearities even between single photons^9,10^, which leads to the realization of a wide range of applications in quantum information processing^11,12^.

A prominent example of the underlying interaction mechanism is the case where the presence of one excited particle can perturb or even prevent the excitation of another by shifting its energy via the interaction between the two Rydberg states. This Rydberg blockade^13^ not only enables rapid saturation at very low light intensities^14–16^, but can also lead to the emergence of strongly correlated many-body states of Rydberg excitations^17,18^. While such correlations can be observed directly on a microscopic level in cold-atom experiments^19,20^, they are more difficult to access in solid-state systems.

Microscopically the Rydberg blockade can be traced back to the asymptotic interaction between neutral particles, such as atoms or excitons, which is dominated by the van der Waals potential that decreases as a simple power law, *V*(*r*) = *C*_6_/*r*^6^, with the inter-particle distance *r*. For electronic ground states, however, the van der Waals coefficient *C*_6_ is typically very small and short-distance exchange effects play an important role in the overall interaction that can often be described in terms of zero-range collisions^21^. The drastic increase of *C*_6_ ~ *n*^11^ with the principal quantum number *n* of excited states, on the other hand, gives rise to exaggerated van der Waals interactions that can be sufficiently strong to even affect the very process of optically generating high-lying Rydberg states, as observed and exploited in cold-atom systems.

In this work, we demonstrate the existence of strongly correlated exciton states by realizing a binary mixture of interacting excitons in high-lying quantum states with different principal quantum numbers via two-color optical excitation in the semiconductor Cu_2_O. Through an adequate choice of Rydberg states and laser intensities, we implement an asymmetric Rydberg blockade^22^ between excitons, where the interactions among identical excitons are of minor importance while the interactions between excitons in different quantum states result in strong spatial correlations and an extended excitation blockade of inter-species exciton pairs. Our experiments exploit this asymmetry to employ one exciton species as a probe for the presence of the other, and reveal direct spectroscopic signatures of the emerging spatial correlations between the two species as well as the distinct power-law character of their mutual interaction.

## Results

### Experimental setting

**Fig. 1:**
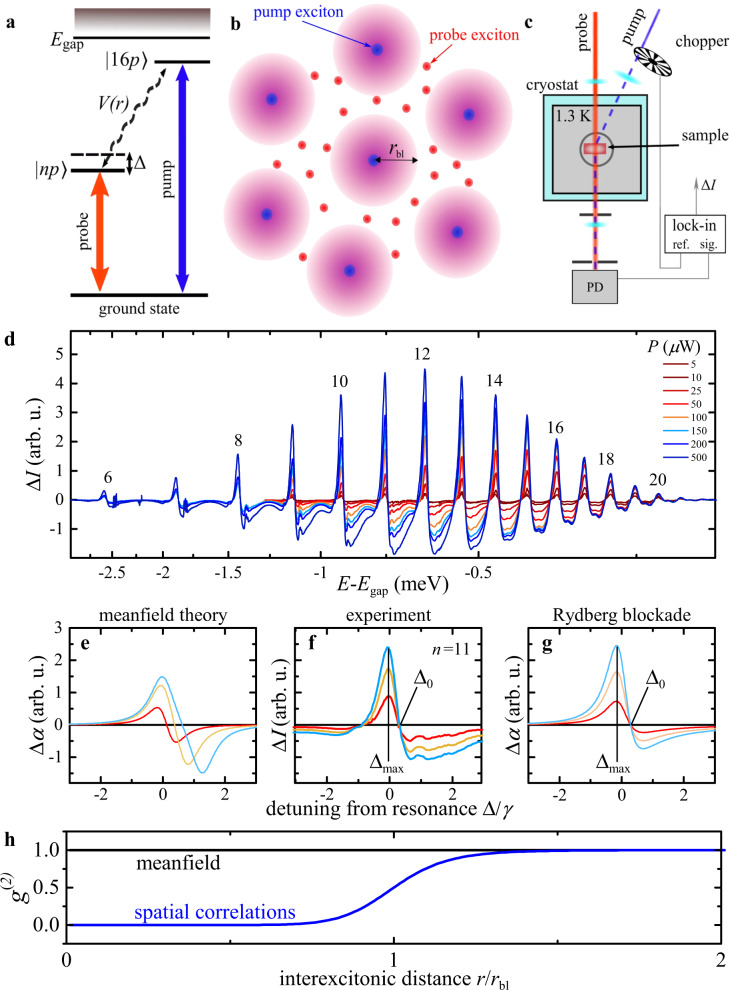
Two-color spectroscopy of excitonic Rydberg series. **a** A weak pump laser (blue) excites excitons in the 16*p* state which interact via the interaction potential *V*(*r*) with *np* excitons that are excited by a weak probe laser (red) at a certain detuning Δ. *E*_gap_ = 2.17208 eV indicates the band gap. **b** These inter-state interactions have a profound effect on the probe-exciton dynamics and tend to block their optical generation within pump-probe distances below the blockade radius *r*_bl_, as indicated by the violet spheres. **c** In our experiment, we modulate the pump beam by an optical chopper and use a lock-in amplifier to lock the recording of the transmitted probe photons (sig.) to the modulation frequency (ref.). In this way, the output signal of the photodiode (PD) that collects the probe photons is directly proportional to the pump-induced differential probe transmission Δ*I*. Examples of the recorded spectra are shown in **d**, which display a series of clear resonances for probe excitons with principal quantum numbers from *n* = 6 to *n* = 20. The increasing signal strength with pump power, *P*, provides indication for the presence of interactions between pump and probe excitons. A common meanfield treatment of such interactions is shown in panel **e** for three increasing interaction strengths. Here, the red line denotes the weakest interaction strength, while the blue line corresponds to the largest interaction strength. However, it fails to reproduce the universal maximum and the isosbestic point observed at fixed laser detunings ${{{\Delta }}}_{\max }$ and Δ_0_, respectively, in the experiment, as indicated in **f** for the *n* = 11 resonance. On the other hand, these features are well explained by a theory that accounts for strong exciton correlations and excitation-blockade effects, as shown in **g**, where again, the red line denotes the weakest and the blue line the largest interaction strength. In **e**–**g**, the detuning is normalized to the linewidth *γ* of the resonance. The underlying spatial correlation function, *g*^(2)^(*r*) between pump and probe excitons, shown in **h**, displays an extended exciton blockade for distances below the blockade radius *r*_bl_, which is on the order of several *μ*m in our experiments.

Our experiments make it possible to probe blockade effects in a semiconductor material at an extreme range of several *μ*m. To this end, we have implemented a two-color excitation scheme, whereby two spectrally-narrow laser beams with different frequencies excite Rydberg excitons with different principal quantum numbers *n* and $n^{\prime}$, respectively (see Fig. 1a). We use a natural Cu_2_O crystal that is cut and polished to a thickness of 30 *μ*m and held at a temperature of 1.3 K. The selection rules associated with the involved band symmetries allow us to excite excitonic *p*-states in the so-called yellow series via single-photon absorption in the optical domain. Hereby, a pump laser generates *p*-state excitons with $n^{\prime} =16$, while another weak probe field with variable frequency creates Rydberg excitons with variable principal quantum numbers *n* = 6, ..., 20 that sense the presence of the pump excitons via their mutual interactions (Fig. 1b). By varying the power of the pump beam, *P*, we can control the density of the pump excitons and monitor their effect on the probe-field absorption around a given probe-exciton resonance.

To achieve the required sensitivity for an accurate measurement of interaction effects, we modulate the pump laser by an optical chopper and detect the transmitted intensity of the probe laser, *I*, with a photodiode that is connected to a lock-in amplifier and locked to the pump modulation frequency (see Fig. 1c and the Methods for details). By choosing a low modulation frequency of 3.33 kHz far below any relevant dynamical frequency scale in the system, we ensure that the resulting signal yields the spectral form of the pump-induced change in the probe transmission Δ*I* ∝ *I*(*P*) − *I*(*P* = 0). Importantly, this approach makes it possible to scan the Rydberg series of the probe excitons while maintaining otherwise stable excitation conditions.

### Differential probe spectra

Figure 1d summarizes the result of such measurements and shows differential probe spectra for a varying power *P* of the pump laser (see Supplementary Note 1 for further details). As the density, *ρ*, of pump excitons grows with *P*, the depicted power dependence carries information about the effects of interactions between pump and probe excitons. A positive signal corresponds to an increased transmission caused by the generated pump excitons, and the observed transmission peaks can be assigned to the Rydberg-state resonances of the probe excitons, as indicated in Fig. 1d. For each individual peak, we observe a linear growth of the signal with *P* but no measurable shift of the position, ${{{\Delta }}}_{\max }$, of the transmission peaks, see closeup in Fig. 1f. Here, Δ is the frequency detuning of the probe laser normalized to the linewidth *γ* of the resonance. At first glance, this comes as a surprise, as an increasing density of pump excitons would be expected to cause a larger shift of the probe-exciton line, similar to the known behavior of quantum-well excitons with short-range interactions^23,24^. In addition, we find that the differential transmission Δ*I* crosses zero at the high-energy side of the resonance at a detuning Δ_0_ that is entirely independent of the pump-laser power, making it an isosbestic point. Indeed all of these features provide strong indication for the emergence of extended spatial correlations during the optical generation of excitons, as we shall see below.

Let us first assume that spatial correlations are insignificant. In this case, the interactions between pump and probe excitons would lead to a simple energy shift, Δ_mf_ = *ρ*∫d**r***V*(*r*), of the probe exciton resonance that is determined by the exciton interaction potential *V*(*r*) and increases linearly with the pump-exciton density *ρ*. Note that such a meanfield treatment describes many aspects of excitons with weak short-range interactions^25^, such as the observed nonlinear spectral properties of semiconductor microcavities^26^, or the fluid-like behavior of exciton-polaritons in such settings^27^. For our experiments, however, a pure meanfield picture fails to capture the essential physics of the exciton dynamics as it predicts a power-dependent position of the maxima and roots of the differential transmission. The meanfield prediction is illustrated in Fig. 1e for three different interaction strengths, and stands in stark contrast to our measurements shown in the middle panel of the same figure.

Such qualitative discrepancies indicate that emerging correlations between the interacting excitons play a significant role during their optical generation. We can explore this further by theoretically considering the correlated excitation dynamics of probe excitons in a background of pump excitons with a density *ρ*. As the interaction, *V*(*r*), between the pump- and probe-excitons shifts the energy of exciton-pair states, it leads to strong spatial correlations following a pump-probe correlation function, that assumes a particularly simple form

1 $${g}^{(2)}(r)=\frac{{\gamma }^{2}/4+{{{\Delta }}}^{2}}{{\gamma }^{2}/4+{[V(r)-{{\Delta }}]}^{2}}$$

to lowest order in the probe intensity (see Supplementary Notes 3 and 4). The function *g*^(2)^(*r*) yields the probability to generate a probe exciton in the vicinity of a pump exciton at a distance *r*. As illustrated in Fig. 1h, this probability vanishes rapidly as the distance between excitons falls below a critical radius *r*_bl_ that is determined by the linewidth *γ* and frequency detuning Δ (see Fig. 1a) of a given Rydberg-state resonance. In our experiments, the range of this exciton blockade can take on remarkably large values of several *μ*m that would significantly affect the probe-beam transmission as expressed by the change of the absorption coefficient

2 $$\alpha ={\alpha }_{0}\left(1-\rho \int \,\frac{(V(r)-2{{\Delta }})V(r)}{{\gamma }^{2}/4+{{{\Delta }}}^{2}}{g}^{(2)}(r){\rm{d}}{\bf{r}}\right)$$

relative to the absorption *α*_0_ in the absence of the pump-beam excitons. For low absorption, the proportionality Δ*I* ∝ Δ*α* = *α*_0_ − *α* affords direct comparisons of our differential transmission measurements with the prediction of Eqs. (1) and (2). As demonstrated in Fig. 1g, our theory for the correlated exciton dynamics indeed reproduces the essential features of our observations. In particular, it yields a maximum and an isosbestic point on the blue side of the differential transmission spectrum that is independent of the pump-beam intensity. This characteristic behavior is also evident from Eq. (2), since the spectral shape of *α*_0_(Δ) − *α*(Δ) is solely determined by the exciton interaction and pair correlation function, while the pump-exciton density merely enters as a linear pre-factor, which reproduces the observed overall linear scaling of the signal with the pump-beam power.

### Rydberg scaling of the interaction

While these characteristic features already provide clear evidence for the Rydberg blockade and emergence of strong exciton correlations, we can also obtain more detailed information about the underlying van der Waals interaction by analyzing the maxima of the differential transmission around each Rydberg-state resonance. To this end, we have recorded the pump-power dependence of the maximum signal strength, which exhibits a linear scaling Δ*I* = *βP* for low pump-beam intensities with a slope $\beta (n,{n}^{\prime})$ that depends on the principal quantum number of both involved Rydberg states (see Supplementary Notes 2 and 5). By evaluating the integral in Eq. (2) for near-resonant excitation, Δ/*γ* ≪ 1, one finds that the slope should scale as $\beta \propto {r}_{{\rm{bl}}}^{3}$. Here, the blockade radius ${r}_{{\rm{bl}}}={\left(\frac{{C}_{6}}{\gamma /2}\right)}^{1/6}$ corresponds to the distance below which the van der Waals interaction $V({r}_{{\rm{bl}}})={C}_{6}/{r}_{{\rm{bl}}}^{6}$ starts to exceed the width of the probe-exciton resonance. By scaling the observed slope with the measured exciton linewidths *γ* and absorption strengths *α*_0_, we can thus probe the state-dependence of the van der Waals coefficient, *C*_6_ ∝ *β*^2^*γ* (see Supplementary Note 5).

**Fig. 2:**
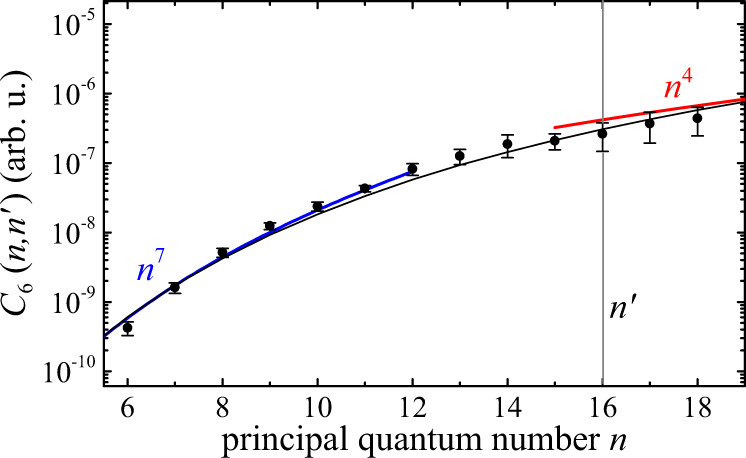
Observed scaling of the van der Waals coefficient. Measured values (black dots) and theoretical prediction following Eq. (3) (black line) for the inter-state van der Waals coefficient ${C}_{6}(n,{n}^{\prime})$, that yields the strength of long-range interactions between excitons with different principal quantum numbers *n* and ${n}^{\prime}$. Here, ${n}^{\prime}=16$ is held fixed while varying *n*, whereby the theory curve has been scaled to match the experiment at *n* = 18. For $n\ll {n}^{\prime}$, theory and experiment give a simple ~ *n*^7^ scaling (blue), which flattens in the opposite limit, when approaching the expected ~ *n*^4^ behavior for $n\gg {n}^{\prime}$. The error bars denote the standard deviation.

Our experimentally determined values are shown in Fig. 2 and indicate a rapid increase of the interaction strength by about three orders of magnitude over the probed range of principal quantum numbers. From the characteristic scaling laws for the level spacings and transition matrix elements of hydrogenic Rydberg states one would expect a characteristic scaling of the interstate van der Waals interaction as (see Supplementary Note 5)

3 $${C}_{6}(n,{n}^{\prime}) \sim \frac{{n}^{4}{({n}^{\prime})}^{4}}{{n}^{-3}+{({n}^{\prime})}^{-3}}=\left\{\begin{array}{l}{n}^{7}{({n}^{\prime})}^{4}\,\text{for}\,n\ll {n}^{\prime}\\ {n}^{4}{({n}^{\prime})}^{7}\,\text{for}\,n\gg {n}^{\prime}\end{array}\right.,$$

which is shown by the black line in Fig. 2. Indeed, our measurements show such a rapid *n*^7^-increase of the interaction for low-lying states ($n\ll {n}^{\prime}$) and also indicate a cross-over to a slower increase, ~*n*^4^, once the excitation level of the probe-exciton exceeds that of the pump excitons at ${n}^{\prime}=16$ ($n\gg {n}^{\prime}$) in accordance with the model prediction.

### Universal spectral shape

**Fig. 3:**
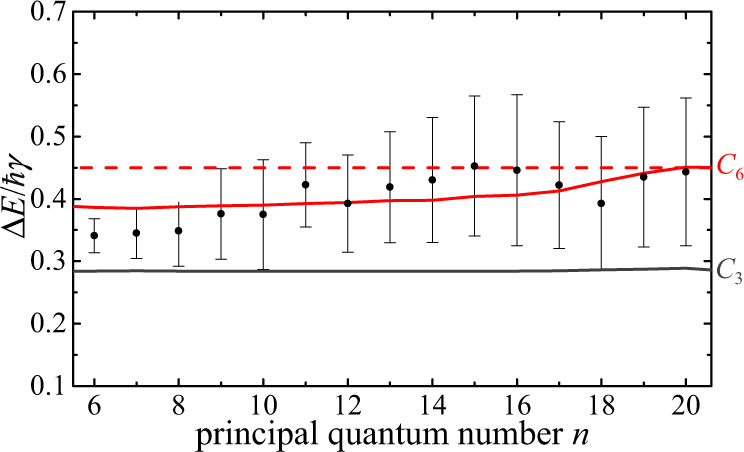
Universal behavior. The scaled difference ${{\Delta }}E/\hslash \gamma =({{{\Delta }}}_{0}-{{{\Delta }}}_{\max })/\gamma$ between the maxima and isosbestic points of the transmission signal does not depend on the pump intensity (see Fig. 1f, g) and is shown here as a function of the principal quantum number *n* of the probe excitons. For homogeneously broadened laser excitation, it becomes a universal quantity that only depends on the type of the interaction and is given by Δ*E*/ℏ*γ* = 0.45 for van der Waals interactions, *V*(*r*) = *C*_6_/*r*^6^, as indicated by the dashed red line. Phonon coupling leads to asymmetric line broadening and causes slight deviations from this behavior (red solid line) in excellent agreement with the measurements shown by the black dots. Other potentials, such as direct dipole-dipole interactions, *V*(*r*) = *C*_3_/*r*^3^, do not match the observations (gray line). The error bars denote the standard deviation.

The peak height of the differential transmission, therefore, demonstrates the power-law scaling of the interaction strength with the principal quantum number of the excitons. In addition, however, its spectral shape also carries spatial information about the power-law decay of the interaction potential as a function of the distance *r* between the excitons. In fact, Eqs. (1) and (2) predict that the scaled energy difference ${{\Delta }}E/\hslash \gamma =({{{\Delta }}}_{0}-{{{\Delta }}}_{\max })/\gamma$ between the positions of the maximum and the isosbestic point of the transmission signal (see Fig. 1c) should depend only on the form and strength of the interaction potential. For pure power-law potentials, Δ*E*/*ℏγ* even becomes independent of the interaction strength and assumes a characteristic universal value for each power-law exponent, which is given by Δ*E*/*ℏγ* = 0.45 for the van der Waals interaction, *V*(*r*) ~ 1/*r*^6^. As shown in Fig. 3, our measurements indeed approach this universal value for high principal quantum numbers *n* of the Rydberg excitons. The spectral line shape of lower lying states is stronger affected by exciton-phonon coupling, which leads to asymmetric broadening that causes small deviations from the universal behavior. These deviations are remarkably well captured by corrections based on Fano-resonance theory^28^ as shown by the red line in Fig. 3 (see Supplementary Note 6).

## Discussion

The combined analysis of our pump-probe measurements thus offer broad insight into the microscopic mechanisms of exciton interactions, and provide a first experimental case for the action of long-range electrostatic van der Waals interactions between excitons in a semiconductor. Being sensitive to the shape and strength of the underlying interaction potential, our scheme makes it possible to discriminate between different types of interactions and clearly excludes direct dipole-dipole interactions with *V*(*r*) ~ 1/*r*^3^ (see Fig. 3). One can explore this capability further by pumping the system above the band gap to create free charges instead of initial excitons (see Supplementary Note 7). The resulting pump-probe spectra give clear evidence for a rapid dynamical recombination of the produced electron-hole plasma into Rydberg-exciton states similar to the behavior of ultracold atomic plasmas^29,30^. Such elementary relaxation processes could become directly accessible to real-time measurements by operating the presented pump-probe approach with short laser pulses. Indeed, this offers an exciting outlook on the developed method, which, when operated below the band gap, would open a new experimental window into the non-equilibrium dynamics of strongly interacting excitons in a semiconductor. Such temporal control could also make advanced studies of quantum mixtures possible, and, for example, enable the investigation of impurity physics and formation of exotic polarons^31^ in mixtures of ground- and Rydberg-state excitons. Generally, the demonstrated ability to enhance interactions and realize spatially extended blockade effects between different exciton states, while maintaining weak intra-state nonlinearities, suggests interesting applications, such as highly efficient few-photon switches^9^ that could be implemented with optical resonators in near-term experiments.

## Methods

### Experimental Setup

We use a two-color pump-probe setup employing two stabilized dye-lasers with a narrow linewidth of 5 neV that serve as pump and probe beams. The studied sample is a Cu_2_O slab with a thickness of *L* = 34 *μ*m cooled down to 1.35 K inside a liquid helium bath and mounted free of strain. Both lasers are in perfect spatial overlap. To assure a homogeneously distributed pump exciton density, the pump laser’s beam waist is set to 300 *μ*m while the probe’s waist is set to 100 *μ*m, both measured at full width at half maximum. The probe power is kept as low as 1 *μ*W to ensure negligible interactions among probe excitons. The pump beam is periodically switched on and off by an optical chopper blade with a frequency of 3.33 kHz. The transmitted probe intensity is detected with a photodiode connected to the signal input (sig.) of a lock-in amplifier (see Fig. 1c). The reference signal (ref.) is provided by the optical chopper and contains the modulation frequency of the pump beam. The lock-in amplifier mixes both signals which yields a frequency-independent part that is proportional to the pump-induced change in the probe beam transmission Δ*I*. Due to the used low modulation frequency this serves as a quasi-CW signal.

## Supplementary information

Supplementary Information

## Data Availability

The datasets generated during and/or analysed during the current study are available from the corresponding author on reasonable request.
