# Supplementary material for: Asymmetric Rydberg blockade of giant excitons in Cuprous Oxide
Source: Nat Commun. 2021 Jun 11;12:3556. doi: 10.1038/s41467-021-23852-z (PMC8196172; doi:10.1038/s41467-021-23852-z)
Supplement: Supplementary file 1 — Supplementary Information [file 41467_2021_23852_MOESM1_ESM.pdf]

Supplementary Information for:
Asymmetric Rydberg blockade of giant excitons in Cuprous Oxide

Julian Heckötter [1], Valentin Walther[2,3], Stefan Scheel[4], Manfred Bayer[1,5], Thomas Pohl[6], and Marc Aßmann[1]

[1]Experimentelle Physik 2, Technische Universität Dortmund, D-44221 Dortmund, Germany
[2]Center for Complex Quantum Systems, Department of Physics and Astronomy, Aarhus University, Ny Munkegade 120, DK-8000 Aarhus C, Denmark
[3]ITAMP, Harvard-Smithsonian Center for Astrophysics, Cambridge, Massachusetts 02138, USA
[4]Institut für Physik, Universität Rostock, Albert-Einstein-Straße 23, D-18059 Rostock, Germany
[5]Ioffe Institute, Russian Academy of Sciences, 194021 St. Petersburg, Russia
[6]Center for Complex Quantum Systems, Department of Physics and Astronomy, Aarhus University, Ny Munkegade 120, DK-8000 Aarhus C, Denmark

Supplementary Note 1: Separation of interaction regimes

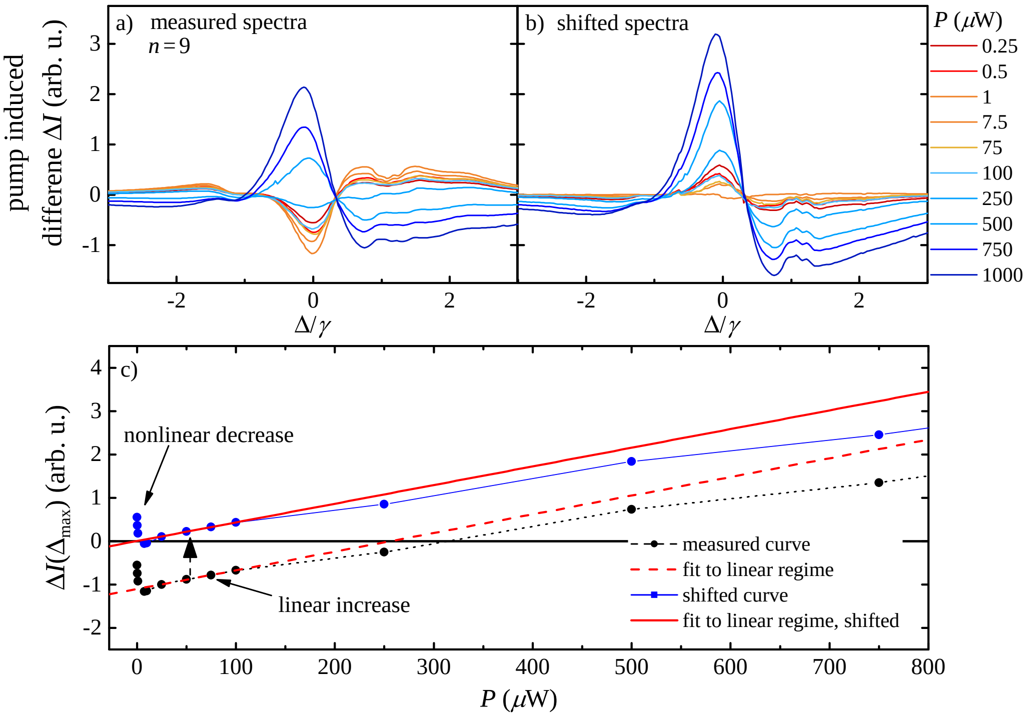

Supplementary Figure 1: Data shift. a) Uncorrected differential transmission spectra around the $n = 9$ resonance. Here, we find negative peak amplitudes for low excitation powers that correspond to a decrease of transmission. b) Corrected spectra around $n = 9$ peak after subtraction of the initial nonlinear decrease of transmission. c) Black dots show the measured maximum signal amplitude of the resonance in panel a) as a function of pump power. The Rydberg interaction is extrapolated linearly towards low intensities (red dashed line). The obtained offset is subtracted from the data resulting in the blue curve. The linear slope does not change (red solid line).

The data presented in Figure 1 in the main text and in Supplementary Figure 4 originally show a defect-induced initial nonlinear decrease of transmission for low pump powers that saturates fast. In order to focus on the Rydberg interaction regime, the data is corrected for this nonlinearity, as described in the following exemplarily for the $n = 9$ resonance. Supplementary Figure 1a) shows the measured differential transmission ΔI around the $n = 9$ resonance for different pump powers P with a focus on the low power range. The pump laser energy is set to the $n' = 16$ resonance.

Starting at low powers (dark red), the transmission around the resonance first decreases, resulting in increasing negative values of the differential transmission ΔI. With growing pump power, this trend is slowed (orange) and finally reversed into enhanced transmission ($\Delta I > 0$, light blue). For a more quantitative discussion, the maximum signal amplitude of the resonance in panel a), $\Delta I(\Delta_{\mathrm{max}})$, is shown as a function of pump power by the black dotted line in panel c). As indicated by the arrow, the nonlinear decrease of transmission can be clearly seen for low pump powers and is found to saturate fast at powers around 5 μW in this particular case. After saturation, the nonlinearity is directly followed by a linear increase of transmission over a wider range of powers. This linear increase is indicated by the red dashed line (see below) and describes the expected linear optical response of the probed state due to Rydberg interactions for low pump beam intensities. At higher powers, the curve flattens and finally saturates.

In order to separate the initial nonlinear decrease of transmission from the exciton interaction regime, the data is extrapolated to zero pump power in the range of linear power dependence, shown by the red dashed line, and the resulting intercept is subtracted from the spectra. The corrected data is given by the blue line with an unchanged slope, given by the red solid line. This procedure is repeated for the whole dataset at every detuning. The resulting corrected spectra around the resonance $n = 9$ are shown in Supplementary Figure 1b).

Supplementary Note 2: Extraction of β

The maximum difference signal at each resonance, $\Delta I(\Delta_{\max})$, exhibits a linear dependence on pump power P in the range of low powers with a characteristic slope β that depends on the principal quantum number n of the probed state. To evaluate the n-dependent scaling of β, the maximum signal amplitude of each resonance is extracted from the data in Figure 1 in the main text as a function of pump power. The data shown for $n = 6 - 8$ at low pump powers (crossed circles) was obtained in a subsequent measurement and is shown for completeness. The obtained values are shown in Supplementary Figure 2 for the states with quantum numbers $n = 6$ to $n = 18$. The linear slope β is obtained by fits to the data within the range of a linear power dependence, as shown by the solid lines. The slope grows with increasing principal quantum number n of the probed state, reflecting a larger interaction strength C_6 for Rydberg excitons with higher n, see Supplementary Eqs. (17) and (18) in Supplementary Note 5. Consequently, the upper end of the linear power range moves to smaller pump powers with increasing n. The blockade volume of an exciton, $V_{\mathrm{bl}} \sim {r_{\mathrm{bl}}}^3$, increases with n. Accordingly, the remaining volume available for absorption decreases faster with increasing pump power for states with higher n and the onset of saturation shifts to lower powers with increasing n as well.

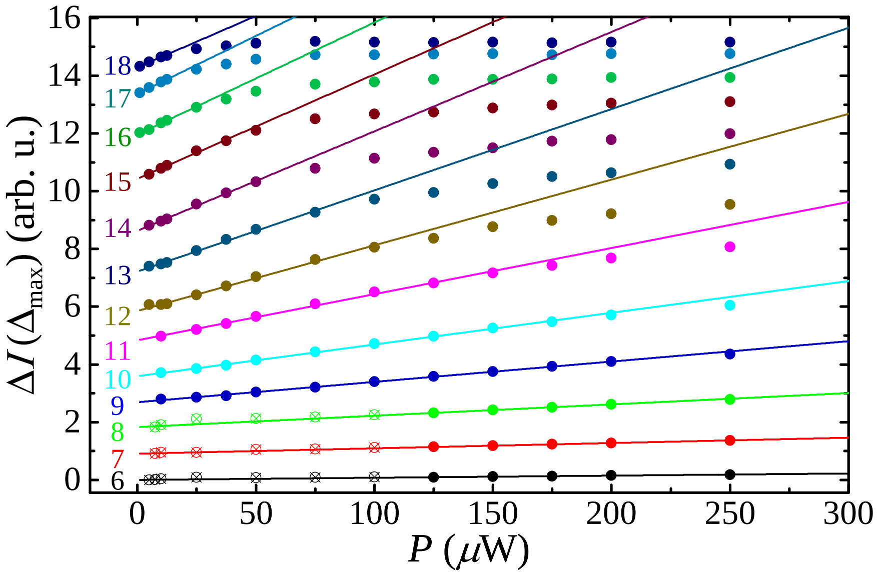

Supplementary Figure 2: Characteristic scaling ($n' = 16$). Maximum differential signal $\Delta I(\Delta_{\max})$ as a function of pump power P for principal quantum numbers $n = 6$ to $n = 18$. The data is obtained from the spectra shown in Figure 1d in the main text (dots) and a second data set focusing on states $n = 6 - 8$ at lower powers (crossed circles). The solid lines show fits with a slope β in the range of a linear dependence on pump power for each resonance. The slope of each fit increases with n reflecting a stronger optical response for states with larger n. Note all curves originate at $\Delta I = 0$, but are shifted vertically for better visibility.

Supplementary Note 3: Exciton correlations

Here, we present details of the theory of the coupled dynamics of pump and probe excitons. Probe excitons, described by $\hat{X}(\mathbf{r})$, are created at an optical coupling rate g from the coherent probe laser of amplitude $\mathcal{E}$ with a detuning given by $\Delta = \omega_{\text{in}} - \omega_{\text{ex}}$. The excitonic resonance has a linewidth γ. Interactions with the pump excitons, described by $\hat{Y}(\mathbf{r})$, are captured by a potential V. We assume that the density of probe excitons is much lower than the pump exciton density, such that interactions among the probe excitons can be neglected. The Heisenberg equation of motion for a probe exciton describes the dynamics

$$\partial_t \hat{X}(\mathbf{r}) = -\frac{\Gamma}{2}\hat{X}(\mathbf{r}) - ig\mathcal{E}(\mathbf{r}) - i\int d\mathbf{r}' V(|\mathbf{r}-\mathbf{r}'|)\hat{Y}^\dagger(\mathbf{r}')\hat{Y}(\mathbf{r}')\hat{X}(\mathbf{r}), \tag{1}$$

where decay and detuning are summarized into the complex linewidth $\Gamma = \gamma - 2i\Delta$. To obtain a solution for the polarization, $\propto \langle \hat{X} \rangle$, we formulate a hierarchy of exciton correlators based on Supplementary Eq. (1) and subsequently solve the steady-state equations

$$\begin{aligned}\partial_t \hat{Y}^\dagger(\mathbf{r}')\hat{Y}(\mathbf{r}')\hat{X}(\mathbf{r}) = &-\frac{\Gamma}{2}\hat{Y}^\dagger(\mathbf{r}')\hat{Y}(\mathbf{r}')\hat{X}(\mathbf{r}) - ig\hat{Y}^\dagger(\mathbf{r}')\hat{Y}(\mathbf{r}')\mathcal{E}(\mathbf{r})\\ &- iV(|\mathbf{r}-\mathbf{r}'|)\hat{Y}^\dagger(\mathbf{r}')\hat{Y}(\mathbf{r}')\hat{X}(\mathbf{r})\\ &- i\int d\mathbf{r}'' V(|\mathbf{r}-\mathbf{r}''|)\hat{Y}^\dagger(\mathbf{r}')\hat{Y}^\dagger(\mathbf{r}'')\hat{X}_p(\mathbf{r}')\hat{X}(\mathbf{r}'')\hat{X}(\mathbf{r}).\end{aligned} \tag{2}$$

In the derivation of Supplementary Eq. (2), we neglect the time dependence of the pump exciton density, as the pump laser is chopped and the pump exciton lifetime is long. The second-to-last term describes the interaction-induced shift inflicted on the probe exciton coherence at $\mathbf{r}$ by the presence of a pump exciton at position $\mathbf{r}'$, while the last term describes simultaneous interactions of a probe exciton with multiple pump excitons. In the limit of low densities relevant for the experiments, we can drop the last term, thus closing the system of equations after taking expectation values

$$\langle \hat{Y}^\dagger(\mathbf{r}')\hat{Y}(\mathbf{r}')\hat{X}(\mathbf{r})\rangle = -i\frac{g\rho(\mathbf{r}')}{\Gamma/2 + iV(|\mathbf{r}-\mathbf{r}'|)}\mathcal{E}(\mathbf{r}), \tag{3}$$

where $\rho(\mathbf{r}') = \langle \hat{Y}^\dagger(\mathbf{r}')\hat{Y}(\mathbf{r}')\rangle$ is the pump exciton density. This leads to the steady-state expression for the exciton coherence

$$\langle \hat{X}(\mathbf{r})\rangle = -\frac{2ig}{\Gamma}\mathcal{E}(\mathbf{r})\left(1 - i\int d\mathbf{r}'\rho(\mathbf{r}')\frac{V(|\mathbf{r}-\mathbf{r}'|)}{\Gamma/2 + iV(|\mathbf{r}-\mathbf{r}'|)}\right). \tag{4}$$

The expectation value for finding a probe exciton in the vicinity of a pump exciton can be calculated analogously from Supplementary Eq. (1)

$$\begin{aligned}\langle \hat{X}^\dagger(\mathbf{r})\hat{Y}^\dagger(\mathbf{r}')\hat{Y}(\mathbf{r}')\hat{X}(\mathbf{r})\rangle &= \frac{2}{\gamma}\Re\left[ig\langle \hat{Y}^\dagger(\mathbf{r}')\hat{Y}(\mathbf{r}')\hat{X}(\mathbf{r})\rangle\mathcal{E}^*(\mathbf{r})\right]\\ &= \frac{g^2\rho^2(\mathbf{r})}{\gamma^2/4 + \Delta^2}g^{(2)}(\mathbf{r},\mathbf{r}')|\mathcal{E}(\mathbf{r})|^2,\end{aligned} \tag{5}$$

where we define the probe-pump correlation function

$$g^{(2)}(\mathbf{r},\mathbf{r}') = \frac{\gamma^2/4 + \Delta^2}{\gamma^2/4 + (V(|\mathbf{r}-\mathbf{r}'|) - \Delta)^2}, \tag{6}$$

as given in Eq. (1) of the main text.

The above theory is qualitatively different from a meanfield model that neglects correlations between the pump and probe excitons. Thus, the latter finds a flat correlation function $g^{(2)}$, as shown in Figure 1h of the main text. In the meanfield, the coherence is directly obtained by factorizing the last term in Supplementary Eq. (1)

$$\langle \hat{X}(\mathbf{r}) \rangle = -i \frac{2g}{\Gamma + 2i\Delta_{\mathrm{mf}}(\mathbf{r})} \mathcal{E}(\mathbf{r}) \tag{7}$$

with the meanfield shift $\Delta_{\mathrm{mf}}(\mathbf{r}) = \int d\mathbf{r}' V(|\mathbf{r} - \mathbf{r}'|)\rho(\mathbf{r}')$.

Supplementary Note 4: Absorption

The propagation of the probe field amplitude $\mathcal{E}(r)$ close to an exciton resonance described by $\hat{X}$ is determined by

$$\partial_t \mathcal{E}(\mathbf{r}) + \frac{c}{\bar{n}} \partial_z \mathcal{E}(\mathbf{r}) = -i \frac{g}{\bar{n}^2} \hat{X}(\mathbf{r}) \tag{8}$$

with the refractive index $\bar{n} = 2.74$ and the speed of light c. Taking expectation values, a closed absorption equation is obtained upon substitution of Supplementary Eq. (4) into Supplementary Eq. (8)

$$\partial_z \mathcal{E}(\mathbf{r}) = -\frac{2g^2}{c\bar{n}\Gamma} \left(1 - i \int d\mathbf{r}' \rho(\mathbf{r}') \frac{V(|\mathbf{r} - \mathbf{r}'|)}{\Gamma/2 + iV(|\mathbf{r} - \mathbf{r}'|)} \right) \mathcal{E}(\mathbf{r}). \tag{9}$$

The absorption coefficient associated with the probe field intensity, $I(\mathbf{r}) = \hbar\omega_{\mathrm{in}} c/\bar{n} |\mathcal{E}(\mathbf{r})|^2$, is then given by

$$\alpha = \alpha_0 \left(1 - \int d\mathbf{r}' \rho(\mathbf{r}') \frac{(V(|\mathbf{r} - \mathbf{r}'|) - 2\Delta) V(|\mathbf{r} - \mathbf{r}'|)}{\gamma^2/4 + \Delta^2} g^{(2)}(\mathbf{r}, \mathbf{r}') \right). \tag{10}$$

The first term describes the standard Lorentzian shape of an exciton resonance in the absence of a pump laser

$$\alpha_0 = \frac{4g^2\gamma}{c\bar{n}(\gamma^2 + 4\Delta^2)}. \tag{11}$$

The second term captures corrections to linear order in the density of pump excitons. We assume for simplicity that this density is constant across the sample, $\rho(\mathbf{r}') = \rho$. The integral boundaries can then be shifted to give the particularly simple form

$$\alpha = \alpha_0 \left(1 - \rho \int \frac{(V(r) - 2\Delta) V(r)}{\gamma^2/4 + \Delta^2} g^{(2)}(r) d\mathbf{r} \right), \tag{12}$$

as given in Eq. (2) of the main text.

The corresponding meanfield absorption is obtained from the coherence given in Supplementary Eq. (7)

$$\alpha_{\mathrm{mf}} = \frac{4g^2\gamma}{c\bar{n}(\gamma^2 + 4(\Delta - \Delta_{\mathrm{mf}})^2)}. \tag{13}$$

In effect, the meanfield approximation shifts the resonance position by Δ_{mf} with respect to the linear response, as can be seen by comparison with Supplementary Eq. (11), and as is illustrated in Figure 1e of the main text.

Supplementary Note 5: Universal absorption shape and scaling of β

The transmitted intensity after propagation through the crystal of length L is given by Beer-Lambert's law $I = I_0 e^{-\alpha L}$. The pump-induced transmission difference is, therefore, directly obtainable from Supplementary Eq. (10)

$$\Delta I = I_0 \left(e^{-\alpha L} - e^{-\alpha_0 L}\right) = I_0 e^{-\alpha_0 L} \left(e^{\alpha_0 \rho L \int \frac{(V(r)-2\Delta)V(r)}{\gamma^2/4+\Delta^2} g^{(2)}(r) d\mathbf{r}} - 1 \right). \quad (14)$$

In the limit of low densities, the exponential in brackets can be expanded giving an expression for

$$\beta = \frac{\Delta I}{P} \approx I_0 e^{-\alpha_0 L} \alpha_0 L \frac{\rho}{P} \int \frac{(V(r) - 2\Delta)V(r)}{\gamma^2/4 + (V(r) - \Delta)^2} d\mathbf{r}. \quad (15)$$

For exciton-exciton interaction of van der Waals type, $V(r) = C_6/r^6$, the integral can be simplified into

$$\beta = I_0 e^{-\alpha_0 L} 4\pi \alpha_0 L \frac{\rho}{P} r_{\text{bl}}^3 \int_0^\infty \frac{(1/\bar{r}^6 - 4\bar{\Delta})1/\bar{r}^6}{1 + (1/\bar{r}^6 - 2\bar{\Delta})^2} \bar{r}^2 d\bar{r}, \quad (16)$$

where we introduced dimensionless variables $\bar{\Delta} = \Delta/\gamma$ and $r = r_{\text{bl}}\bar{r}$ with the blockade radius $r_{\text{bl}} = \sqrt[6]{\frac{C_6}{\gamma/2}}$. This shows that the transmission difference ΔI, or β, have a universal shape as a function of $\bar{\Delta}$, independent of the strength of the interaction. In particular, this proves the fixed position of the isosbestic point and maximum across all principal quantum numbers as discussed in the main text and displayed in Figure 1g therein. We note that the linear absorption factor, represented by the exponential in Supplementary Eq. (16), technically also influences the curve shape. However, since our main interest is in the pump-induced transmission maximum close around $\bar{\Delta} = 0$ and the isosbestic point, this effect is small and neglected in the following.

Supplementary Eq. (16) also isolates the dependence on the principal quantum number n. In the limit of radiative scaling, optical dipole coupling scales as $g \sim n^{-\frac{3}{2}}$ and the linewidth scales as $\gamma \sim n^{-3}$, leaving a constant linear absorption α_0. Thus, the n dependence of β for a given n' mirrors that of the blockade radius

$$\beta(n) \sim r_{\text{bl}}^3 = \sqrt{\frac{C_6}{\gamma/2}}. \quad (17)$$

The van der Waals coefficient's dependence on probe and pump principal quantum number can be estimated from second-order perturbation theory [1] from the probe [pump] exciton's dipole moment $d_{\text{pr}}(n) \sim n^2$ [$d_{\text{pu}}(n') \sim (n')^2$] and the pair state Förster energy $\delta(n, n') \sim n^{-3} + (n')^{-3}$ as

$$C_6(n, n') \sim \frac{d_{\text{pr}}^2 d_{\text{pu}}^2}{\delta} \sim \frac{n^4 (n')^4}{n^{-3} + (n')^{-3}} = \begin{cases} n^7 (n')^4 & \text{for } n \ll n' \\ n^4 (n')^7 & \text{for } n \gg n' \end{cases}. \quad (18)$$

The optical coupling rates g and linewidths γ of exciton absorption lines in Cu_2O are known to deviate from ideal scalings $\sim n^{-3}$, in particular at high n [2]. Most prominently, this results in n-dependent absorption strengths $\alpha_0 \sim g^2/\gamma$ in linear absorption spectra (see Supplementary Eq. (11)). The non-monotonic behavior of α_0 with n results in a tent-like envelope that can directly be observed in Figure 1d in the main text at the lowest pump intensities.

To obtain C_6 from experiment from the measured $\beta(n)$, the constant α_0 in Supplementary Eq. (16) is substituted by measured values $\alpha_{0,\mathrm{exp}} \sim g_{\mathrm{exp}}^2/\gamma_{\mathrm{exp}}$ extracted from absorption spectra

$$\beta(n) \sim \alpha_{0,\mathrm{exp}} L e^{-\alpha_{0,\mathrm{exp}} L} r_{\mathrm{bl}}^3 = \sqrt{\frac{C_6}{\gamma_{\mathrm{exp}}/2}} \, . \tag{19}$$

Finally, C_6 is then obtained by $C_6(n, n') \sim \tilde{\beta}^2 \gamma_{\mathrm{exp}}$, with $\tilde{\beta} = \frac{\beta}{\alpha_{0,\mathrm{exp}} L e^{-\alpha_{0,\mathrm{exp}}}}$, where β are the linear slopes of fits to the data, as shown in Supplementary Figure 2. This gives the experimental interaction strengths shown in Figure 2 of the main text.

Supplementary Note 6: Asymmetry from phonon background

The absorption lines of the yellow series in Cu_2O show an asymmetric curve shape due to an interference with a spectrally broad phonon background [3]. This affects the shape of the pump-induced difference absorption as we show below. In the excitation process, an incident photon can either couple to the phonon background, described by a set of operators $\hat{X}_{\mathbf{k}}$, directly or first to a Rydberg exciton and then to the phonon continuum, thus creating a Fano resonance [4]. We capture this by expanding Supplementary Eq. (1) and adding a phonon equation of motion

$$\partial_t \hat{X}(\mathbf{r}) = -\frac{\Gamma}{2}\hat{X}(\mathbf{r}) - ig\mathcal{E}(\mathbf{r}) - i\sum_{\mathbf{k}} h_{\mathbf{k}}\hat{X}_{\mathbf{k}} - i\int d\mathbf{r}' V(|\mathbf{r}-\mathbf{r}'|)\hat{Y}^\dagger(\mathbf{r}')\hat{Y}(\mathbf{r}')\hat{X}(\mathbf{r}) \tag{20}$$

$$\partial_t \hat{X}_{\mathbf{k}}(\mathbf{r}) = -\frac{\Gamma_{\mathbf{k}}}{2}\hat{X}_{\mathbf{k}}(\mathbf{r}) - ig_{\mathbf{k}}\mathcal{E}(\mathbf{r}) - ih_{\mathbf{k}}\hat{X}(\mathbf{r}), \tag{21}$$

where $g_{\mathbf{k}}$ denotes the optical coupling rate to the phonons, $h_{\mathbf{k}}$ the exciton-phonon coupling and $\Gamma_{\mathbf{k}} = \gamma_{\mathbf{k}} - 2i\Delta_{\mathbf{k}}$ the complex phonon linewidth with linewidth $\gamma_{\mathbf{k}}$ and detuning $\Delta_{\mathbf{k}} = \omega_{\mathrm{in}} - \omega_{\mathbf{k}}$. The light propagation is modified into

$$\partial_t \mathcal{E}(\mathbf{r}) + \frac{c}{\bar{n}}\partial_z \mathcal{E}(\mathbf{r}) = -i\frac{g}{\bar{n}^2}\hat{X}(\mathbf{r}) - i\sum_{\mathbf{k}} \frac{g_{\mathbf{k}}}{\bar{n}^2}\hat{X}_{\mathbf{k}}(\mathbf{r}). \tag{22}$$

We start by considering the non-interacting system, $V = 0$. In the steady-state, we can solve for the continuum operators

$$\langle \hat{X}_{\mathbf{k}}(\mathbf{r})\rangle = \frac{2}{\Gamma_{\mathbf{k}}}\left[-ig_{\mathbf{k}}\mathcal{E}(\mathbf{r}) - ih_{\mathbf{k}}\langle \hat{X}(\mathbf{r})\rangle\right]. \tag{23}$$

Across a single exciton resonance, the phonon states can for simplicity be assumed as flat ($g_{\mathbf{k}} =$ const. and $h_{\mathbf{k}} =$ const.) and dense, allowing the sums to be approximated by integrals and evaluated using Dirac's identity

$$\lim_{\epsilon \searrow 0} \int f(x)\frac{1}{x - i\epsilon} = i\pi\int f(x)\delta(x) + \mathcal{P}\int \frac{f(x)}{x}. \tag{24}$$

In the limit $\gamma_{\mathbf{k}} \to 0$, this renders

$$\sum_{\mathbf{k}} h_{\mathbf{k}}\langle \hat{X}_{\mathbf{k}}(\mathbf{r})\rangle = -i\pi\bar{h}\bar{g}\mathcal{E}(\mathbf{r}) - i\pi\bar{h}^2\langle \hat{X}(\mathbf{r})\rangle, \tag{25}$$

where we absorbed the integration constants and the factor π into $\bar{h}$ and, similarly, $\bar{g}$ (that have units $\sim \sqrt{\text{energy}}$). If the principal value integral does not vanish, it can also be absorbed into the barred constants. The polarization can be solved from Supplementary Eq. (20)

$$\langle \hat{X}(\mathbf{r}) \rangle = \frac{-ig - \bar{h}\bar{g}}{\frac{\Gamma}{2} + \bar{h}^2} \mathcal{E}(\mathbf{r}), \tag{26}$$

giving rise to an asymmetric linear absorption spectrum

$$\alpha_{\text{asym}} = \frac{2}{c\bar{n}} \frac{(g^2 - (\bar{g}\bar{h})^2)(\gamma/2 + \bar{h}^2) + 2g\bar{g}\bar{h}\Delta}{(\bar{h}^2 + \gamma/2)^2 + \Delta^2} + \frac{2}{c\bar{n}} \bar{g}^2. \tag{27}$$

To simplify the expression we introduce $\bar{\gamma}/2 = \gamma/2 + \bar{h}^2$, the asymmetry parameter $Q = \frac{\bar{g}\bar{h}}{g}$ as well as the constant background absorption coefficient $\alpha_{\text{bg}} = \frac{2}{c\bar{n}} \bar{g}^2$ and exploit that the excitation rate is larger than the indirect rate $g \gg \bar{g}\bar{h}$

$$\alpha_{\text{asym}} = \frac{4g^2}{c\bar{n}} \frac{\bar{\gamma} + 4Q\Delta}{\bar{\gamma}^2 + 4\Delta^2} + \alpha_{\text{bg}}. \tag{28}$$

We recognize a constant absorption term from the background as well as the plain exciton absorption from Supplementary Eq. (11). The cross term originates from the interference between direct and indirect excitation of the background and produces an asymmetric lineshape. This shape corresponds to previous descriptions of the asymmetry in Cu_2O [5, 6, 7]. The linear spectra are used to extract Q from experiment.

The interacting system can be solved straightforwardly, following the approach outlined in Supplementary Notes 3-4 and giving the polarization

$$\langle \hat{X}(\mathbf{r}) \rangle = \frac{-2ig(1 - iQ)}{-\bar{\gamma} + 2i\Delta} \left[1 - i \int d\mathbf{r}' \rho(\mathbf{r}') \frac{V(|\mathbf{r} - \mathbf{r}'|)}{\frac{\bar{\gamma}}{2} - i\Delta + iV(|\mathbf{r} - \mathbf{r}'|)} \right] \mathcal{E}(\mathbf{r}). \tag{29}$$

The absorption coefficient, here for flat densities, is readily obtained by inserting Supplementary Eqs. (29) and (23) into Supplementary Eq. (22)

$$\alpha = \alpha_{\text{asym}} + \frac{2}{c\bar{n}} \Re \left[\frac{2ig^2 (1 - iQ)^2}{-\bar{\gamma} + 2i\Delta} \rho \int \frac{V(r)}{\frac{\bar{\gamma}}{2} - i\Delta + iV(r)} d\mathbf{r} \right]. \tag{30}$$

A non-vanishing asymmetry parameter, $Q \neq 0$, thus clearly leads to quantitative changes of the absorption features (Supplementary Figure 3). In particular, the positions of the maximum and the isosbestic point depend on Q. For van der Waals interactions, the difference between these points decreases with Q. For direct dipole-dipole interactions of the form $V(r) = C_3/r^3$, this difference remains almost constant with Q. The corresponding predictions based on the experimental values of Q are shown in Figure 3 of the main text. We remark that the integral in Supplementary Eq. (30) for dipole-dipole interactions has a logarithmic divergence for large r, as can be seen by introducing a sufficiently large R_1

$$\int \frac{V(r)}{\frac{\bar{\gamma}}{2} - i\Delta + iV(r)} d\mathbf{r} \approx 4\pi \left[\int_0^{R_1} \frac{V(r) r^2}{\frac{\bar{\gamma}}{2} - i\Delta + iV(r)} dr + \lim_{R_2 \to \infty} \frac{C_3}{\frac{\bar{\gamma}}{2} - i\Delta} \ln \left(\frac{R_2}{R_1} \right) \right]. \tag{31}$$

However, the nonlinearity is dominated by the second term whose shape as a function of Δ is determined by

$$\Re \left[-i \frac{(1 - iQ)^2}{(\frac{\bar{\gamma}}{2} - i\Delta)^2} \right]. \tag{32}$$

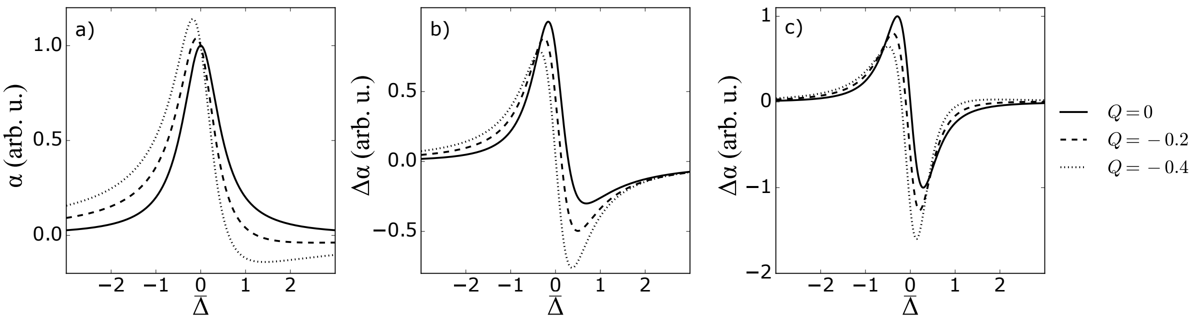

Supplementary Figure 3: Fano asymmetry. a) Linear absorption spectrum as a function of $\bar{\Delta} = \Delta/\bar{\gamma}$ for three different asymmetry parameters Q. The curves are normalized to the maximum of the Lorentzian profile ($Q = 0$). b) Normalized pump-induced differential absorption as a function of the asymmetry parameter Q for van der Waals interactions ($\sim r^{-6}$), curves matching panel a). Positions of maximum and isosbestic point are red-shifted with increasing asymmetry but their difference decreases. c) Same as in b) but for direct dipole-dipole interactions ($\sim r^{-3}$).

Supplementary Note 7: Relation to exciton-in-plasma studies

Strong optical excitation of the crystal by a pump laser beam above the band gap unavoidably leads to the creation of a density of free electron-hole pairs that may additionally influence the exciton states as studied in Ref. [8]. Compared to Ref. [8], we restrict our analysis to very low pump powers, where the differential transmission spectra are linear in the applied pump power. In order to estimate the impact of such an electron-hole plasma on the observed spectra we show a second set of differential transmission spectra recorded with a pump laser energy at 2.20 eV, i.e., 28 meV above the band gap. We repeat the same analysis as in the case of resonant pumping of Rydberg excitons (main text). Supplementary Figure 4a) shows the recorded spectra for pump powers from 0.5 to 500 μW.

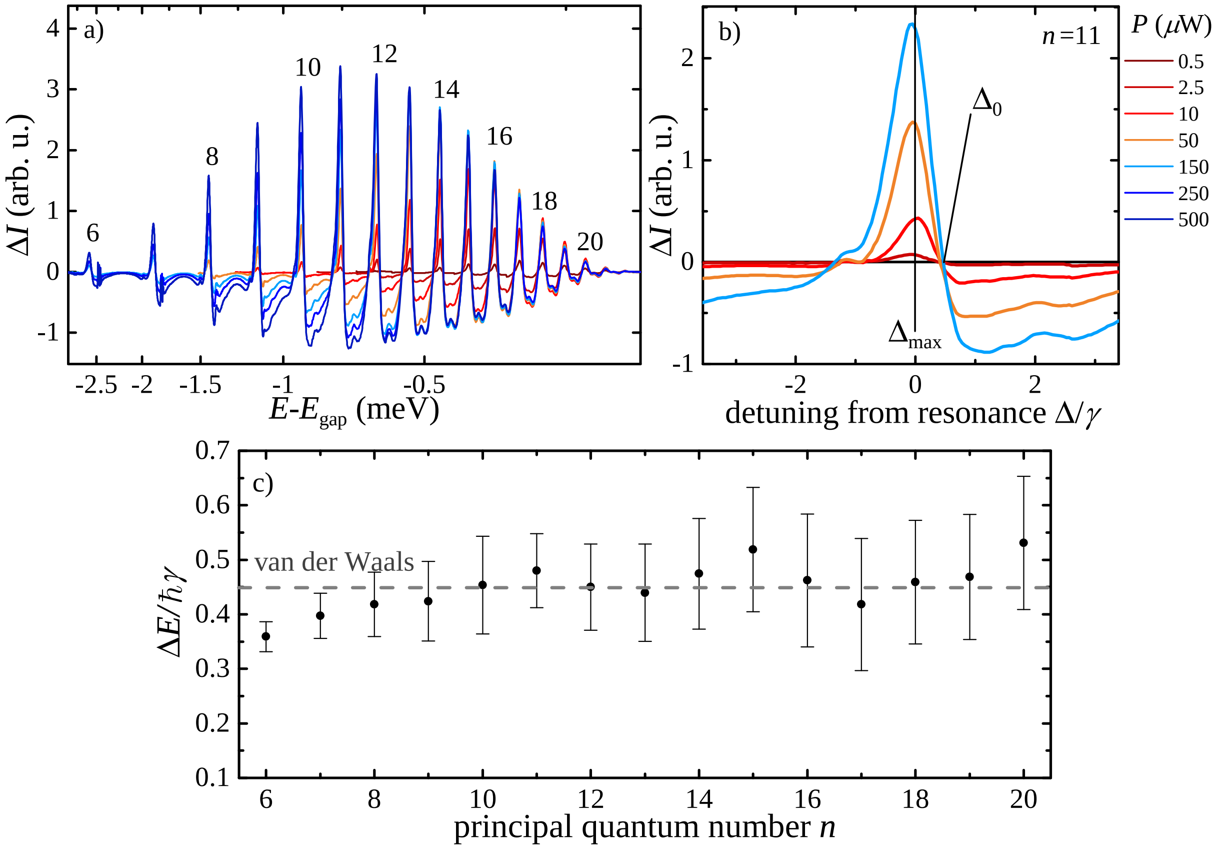

Supplementary Figure 4: Differential signal (plasma). a) Differential transmission ΔI for excitons $n = 6$ up to $n = 21$, recorded with a pump laser energy fixed above the band gap. The pump power P is increased from 0.5 to 500 μW. b) Closeup of the $n = 11$ resonance shown for pump powers in the range of linear power dependence. Also here, the curve shape is universal and exhibits a fixed extreme point at Δ_{max} and an isosbestic point on the high-energy side at Δ_0, where the probe absorption is independent of the pump intensity. c) The experimentally obtained ratio $\Delta E/\hbar\gamma$ is comparable to the theoretical value of 0.45 (grey dashed line), that is predicted for the van der Waals potential. The error bars denote the standard deviation.

First, we investigate the spectral features of the recorded resonances. At first glance, there are no major differences compared to the pump scenario shown in Figure 1d of the main text. We again find a non-shifting maximum growing with increasing pump power and an isosbestic point

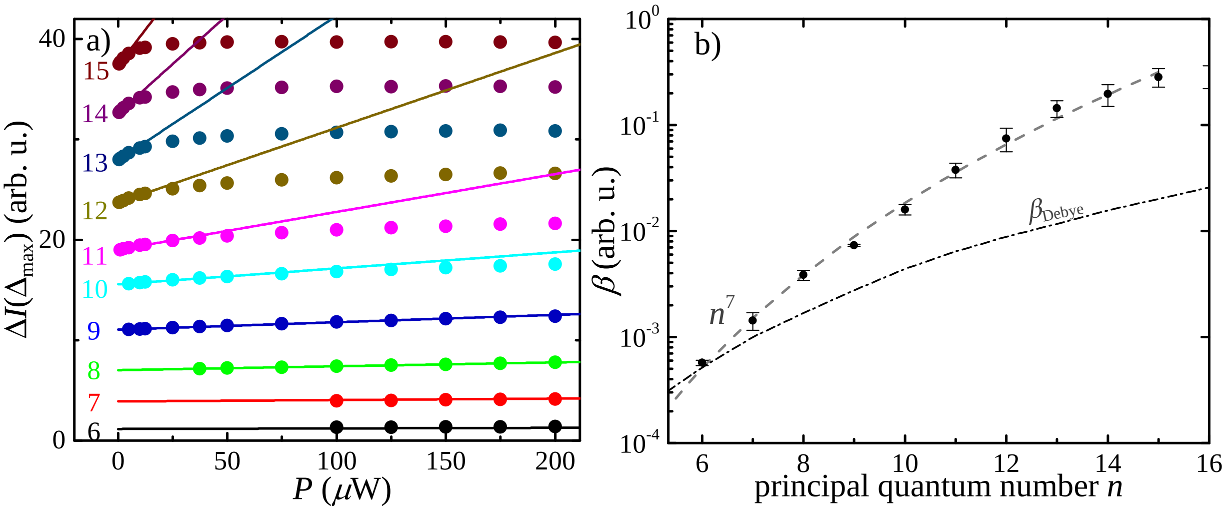

Supplementary Figure 5: Characteristic scaling (plasma). a) Maximum differential signal $\Delta I(\Delta_{\max})$ as a function of pump power P for principal quantum numbers $n = 6$ to 15. The pump laser energy is set above the band gap. The solid lines show fits with a slope β in the range of a linear dependence on pump power for each resonance. Note that the curves originate at $\Delta I = 0$, but are shifted vertically for better visibility. b) Experimental scaling of β as a function of principal quantum number n. The observed scaling follows an n^7 dependence in the range from $n = 6$ to 15 as indicated by the grey dashed line. The black dashed-dotted line shows the scaling of β_{Debye} predicted by the Debye model. It is much weaker than observed in the experiment. The curves are shifted by an arbitrary value to coincide at $n = 6$ for comparison. The error bars denote the standard deviation.

at Δ_0 on the high-energy side, as shown exemplarily for the $n = 11$ resonance in Supplementary Figure 4b). These characteristic signatures result solely from power-law interaction potentials and thus stand in contradiction to a possible dynamical screening induced by a plasma, which can be described to first approximation by a Debye potential [9] $V(r) \sim e^{-\kappa r}/r$. Further, we also determine the universal quantity $\Delta E/\hbar\gamma$ for each resonance and find values that are comparable to the expectations given by the van der Waals interaction of 0.45, as shown in Supplementary Figure 4c). These observations cannot be explained by dynamical screening.

Following the analysis in the main text, we now evaluate the power dependence of the maxima of each resonance in order to determine a characteristic scaling law of the slope β. For the states from $n = 6$ to $n = 15$, we find a pump power regime of linearly increasing maxima, whose range decreases drastically with n (Supplementary Figure 5a)). Remarkably, the experimentally determined linear slope β shows a strong increase as a function of principal quantum number n scaling approximately as n^7 for low and intermediate states (Supplementary Figure 5b), grey dashed line). From the Debye model, we numerically extract a pump power dependence of the matrix elements $g^2 \approx g_0^2 + g_1^2 P$ with $g_1^2 \sim n^{0.85}$. This implies a scaling of $\beta \propto e^{-\alpha_0 L}\frac{g_1^2}{\gamma}$ to lowest order in P. The radiative expectation on the scaling of $\beta \sim n^{3.85}$ is corrected by the experimental values of both α_0 and γ in Supplementary Figure 5b), demonstrating that the Debye model significantly underestimates the observed slope of β.

Interestingly, the observed scaling is even slightly steeper than the one expected for van der Waals interactions with resonantly excited Rydberg pump excitons at fixed n'. In this case the scaling for low n is $\beta(n, n) \propto n^5$, cf. Supplementary Eqs. (17) and (18). The stronger optical response observed in the experiment may be explained by fast relaxation of free charges into an

unknown distribution of excitons with different principal quantum numbers and angular momenta [10, 11].

In conclusion, also for above-band-gap excitation we find characteristic spectral signatures and an n-dependent scaling of the signal strength that are in accordance with van der Waals-type interaction between excitons created by fast relaxation of free electron-hole pairs.

Supplementary References

[1] Walther, V., Krüger, S. O., Scheel, S. & Pohl, T. Interactions between Rydberg excitons in Cu_2O. *Phys. Rev. B* **98**, 165201 (2018).

[2] Kazimierczuk, T., Fröhlich, D., Scheel, S., Stolz, H. & Bayer, M. Giant Rydberg excitons in the copper oxide Cu_2O. *Nature* **514**, 343–347 (2014).

[3] Schöne, F., Stolz, H. & Naka, N. Phonon-assisted absorption of excitons in Cu_2O. *Phys. Rev. B* **96**, 115207 (2017).

[4] Fano, U. Effects of Configuration Interaction on Intensities and Phase Shifts. *Phys. Rev.* **124**, 1866–1878 (1961).

[5] Toyozawa, Y. Interband effect of lattice vibrations in the exciton absorption spectra. *J. Phys. Chem. Solids* **25**, 59–71 (1964).

[6] Jolk, A. & Klingshirn, C. F. Linear and Nonlinear Excitonic Absorption and Photoluminescence Spectra in Cu_2O: Line Shape Analysis and Exciton Drift. *phys. stat. sol. (b)* **206**, 841–850 (1998).

[7] Ueno, T. On the Contour of the Absorption Lines in Cu_2O. *J. Phys. Soc. Jpn* **26**, 438–446 (1969).

[8] Heckötter, J. *et al.* Rydberg Excitons in the Presence of an Ultralow-Density Electron-Hole Plasma. *Phys. Rev. Lett.* **121**, 097401 (2018).

[9] Kremp, D., Schlanges, M. & Kraeft, W.-D. *Quantum Statistics of Nonideal Plasmas.* Springer Series on Atomic, Optical, and Plasma Physics (Springer, Berlin, 2005).

[10] Takahata, M. & Naka, N. Photoluminescence properties of the entire excitonic series in Cu_2O. *Phys. Rev. B* **98**, 195205 (2018).

[11] Killian, T. C. *et al.* Formation of Rydberg Atoms in an Expanding Ultracold Neutral Plasma. *Phys. Rev. Lett.* **86**, 3759–3762 (2001).
